# Use of Time-Resolved Fluorescence to Monitor Bioactive Compounds in Plant Based Foodstuffs

**DOI:** 10.3390/bios5030367

**Published:** 2015-06-26

**Authors:** M. Adília Lemos, Katarína Sárniková, Francesca Bot, Monica Anese, Graham Hungerford

**Affiliations:** 1 Food & Life Sciences, School of Science, Engineering and Technology, University of Abertay Dundee, Bell Street, Dundee DD1 1HG, UK; E-Mails: a.lemos@abertay.ac.uk (M.A.L.); ksarnikova@yahoo.com (K.S.); 2 Department of Food Science, University of Udine, Via Sondrio 2/A, 33100 Udine, Italy; E-Mails: bot.francesca@spes.uniud.it (F.B.); monica.anese@uniud.it (M.A.); 3 HORIBA Jobin Yvon IBH, 133 Finnieston Street, Glasgow G3 8HB, UK

**Keywords:** anthocyanin, betalain, chlorophyll, curcumin, fluorescence lifetime, lycopene

## Abstract

The study of compounds that exhibit antioxidant activity has recently received much interest in the food industry because of their potential health benefits. Most of these compounds are plant based, such as polyphenolics and carotenoids, and there is a need to monitor them from the field through processing and into the body. Ideally, a monitoring technique should be non-invasive with the potential for remote capabilities. The application of the phenomenon of fluorescence has proved to be well suited, as many plant associated compounds exhibit fluorescence. The photophysical behaviour of fluorescent molecules is also highly dependent on their microenvironment, making them suitable probes to monitor changes in pH, viscosity and polarity, for example. Time-resolved fluorescence techniques have recently come to the fore, as they offer the ability to obtain more information, coupled with the fact that the fluorescence lifetime is an absolute measure, while steady state just provides relative and average information. In this work, we will present illustrative time-resolved measurements, rather than a comprehensive review, to show the potential of time-resolved fluorescence applied to the study of bioactive substances. The aim is to help assess if any changes occur in their form, going from extraction via storage and cooking to the interaction with serum albumin, a principal blood transport protein.

## 1. Introduction

### 1.1. Interest in Studying Bioactive Compounds

The study of compounds that exhibit antioxidant activity has recently received much interest in the food industry because of their potential health benefits. This has led to research into their extraction for use as additives to other foods, both for preservation and health reasons, as well as continuing studies of their influence in their native foodstuffs. Generally speaking, these antioxidants are non-nutrient components of food and have been assessed as exhibiting antioxidant activity *in vitro*. These phytochemicals can be responsible for colour, taste and smell of food plants. Foods from plant origin contain a large group of compounds, which are produced by the plant as secondary metabolites, such as polyphenolics. These compounds, are divided into the following main classes; flavonoids, phenolic acids, tannins, stilbenes and lignans [1]. Polyphenolic compounds have been associated with health benefits because of their anti-oxidant, anti-inflammatory, anti-microbial activity [2,3]. It is also known that the consumption of polyphenol rich foods can decrease the risk of chronic diseases, such as cardiovascular diseases, type 2 diabetes and cancer [2,4]. There are other compounds, such as carotenoids [5] and betalains [6], that have also been shown to exhibit antioxidant activity and the consumption of foods containing them has produced interest with researchers in the food industry. This is largely because of their anti-inflammatory properties [7,8] and their potential to give health benefits [9,10] against diseases, such as cancer and cardiovascular disease [11,12], which has given rise to investigation into their use as food additives and preservatives. There is, therefore, a need to be able to monitor these compounds *in situ* as well as if they are extracted for changes that may adversely affect their usage. This includes from their initial growth in the field through any industrial or domestic process (extraction, cooking *etc.*) to their interaction inside the body.

### 1.2. Application of Time-Resolved Fluorescence

**Figure 1:**
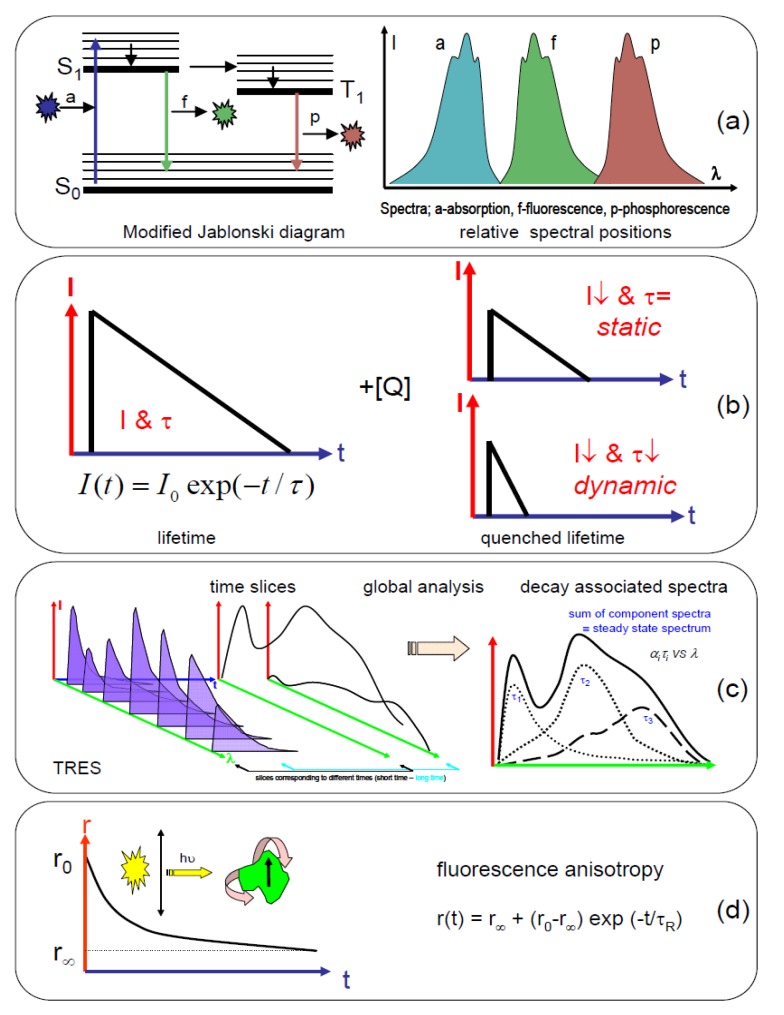
Graphical depiction of time-resolved measurement methods and an indication of the information obtainable. (**a**) A modified Jablonski diagram, plus a relative indication of the wavelength positions of the absorption, fluorescence and phosphorescence spectra; (**b**) Representation of a decay (log intensity scale) and variation of lifetime (τ, indicated by gradient of decay) and intensity (I) on addition of a quencher ([Q]); (**c**) Time-resolved emission spectra (TRES) illustrating time slicing at two points and production of decay associated spectra; (**d**) Representation of fluorescence anisotropy, which can provide the initial anisotropy (r_0_), the limiting anisotropy (r_∞_) and the rotational correlation time (τ_R_).

Since many of these antioxidant compounds either exhibit fluorescence or influence other compounds that emit fluorescence, this optical phenomenon is a promising technique by which to investigate them. The fluorescence process is usually depicted graphically by a Jablonski diagram and Figure 1a gives this type of illustration. There are several texts detailing fluorescence and measurement methods e.g., [13,14], so it will only be examined briefly here. Generally speaking, light (absorbed on the fs timescale), if of the correct energy, can rise from a molecule (fluorophore) to an excited state, from which it returns to the ground state by emission of light (fluorescence). This fluorescence typically occurs on the ps to ns timescale and is shifted to longer wavelengths than the excitation, as the molecule in the excited state can lose energy to its microenvironment. Fluorescence is in fact a multiparameter signal, which depends on factors such as wavelength, time, polarisation and position. Steady state techniques usually involve the measurement of spectra (Figure 1a) giving information concerning energy, while time-resolved methods relate to the length of time that a molecule spends in the excited state. From the excited state, it is possible for the molecule to return to the ground state vibrationally (non-radiative) or by emission of a photon (radiative), and these quantities can help determine the quantum yield of fluorescence. There is also the possibility for intersystem crossing to occur to the triplet state, which can give rise to phosphorescence. This occurs at a longer wavelength (lower energy) and longer timescale (μs to seconds), but requires initial excitation into the singlet state.

The decay of the excited state is usually exponential (Figure 1b) and the fluorescence lifetime is given by τ, which is the time it takes for the fluorescence to decay to 1/e of its initial value. One of the major advantages of using the fluorescence lifetime is the fact that it is an absolute measurement, unlike the steady state intensity, which is a relative measurement. The steady state measurement usually provides an intensity reading with wavelength which will depend, in part, on the excitation intensity and number of fluorescing molecules. The fluorescence lifetime is an intrinsic molecular property and, within certain constraints, independent of concentration. This means that changes in concentration, whether caused by photobleaching or diluting/concentrating the sample, would not affect the lifetime value. This is in contrast to a steady state measurement, where a change in intensity of the recorded emission would be observed. Generally, it is accepted that time-correlated single-photon counting (TCSPC) [15,16] is the most sensitive method by which to obtain the fluorescence lifetime. Using the fluorescence lifetime, there is an implication that a measurement taken on one piece of equipment at a certain time and place should be capable of being repeated later elsewhere (provided the sample is stable and measurement conditions are preserved). Fluorescence is an ideal nanoscale probe, as the fluorescence decay take places on the nanosecond timescale and it can be influenced by molecular processes occurring on the nanometre range. The emission of a fluorophore can be highly influenced by its environment or the presence of other interacting molecules, which can affect the non-radiative rate constant. Thus, the fluorescence lifetime is useful in elucidating;

- changes in the nanoenvironment
  ○ (viscosity, pH, polarity, salvation)
- size and shape of molecules
- molecular interactions
- inter- and intramolecular distances
- kinetic and dynamic rates
- resolution of molecular mixtures

The extra specificity of the fluorescence lifetime allows easy discrimination against scattered excitation and background fluorescence. Determination of Förster resonance energy transfer (FRET) is much simpler using the fluorescence lifetime, as are quenching and fluorescence anisotropy measurements; allowing more parameters to be recovered. However, it should be remembered that the use of steady state and time-resolved techniques are complementary in elucidating as much of the multiparametric fluorescence signature as possible.

In TCSPC measurements, a log intensity axis is commonly used, as the decay appears linear, making data interpretation easier. Interactions can lead to quenching of the fluorescence, which can be either dynamic (change in lifetime and intensity) or static (change in intensity only). This can occur by molecular binding, electron or energy transfer (e.g., FRET) or another environmental change. The exact emission behaviour depends on the individual fluorophore. If more than one excited state is present then the decay can become multiexponential, *i.e.*, the states have different lifetimes. Time-resolved emission spectra (TRES) are useful in the resolution of emitting species which have different lifetimes and overlapping spectral properties. A 3-D surface of intensity-time-wavelength can be obtained, as depicted in Figure 1c. Analysis can be performed by simply taking slices at different times to see the evolution of the spectral shape. It is also possible to perform a global analysis to obtain the lifetimes from the fluorescing species and to further analyse this to recover decay associated spectra. These are spectra relating to the different lifetimes. Another commonly used technique is the measurement of fluorescence anisotropy, Figure 1d. This polarisation measurement is commonly used to show viscosity information, although by the use of molecular rotors a simpler lifetime measurement can also be employed [17,18]. It is also the best way to elucidate homo-FRET and can be used to determine molecular size. Steady state anisotropy only provides one measure (r), while time-resolved enables greater information (*i.e.*, initial anisotropy, r_0_; limiting anisotropy, r_∞_; rotational correlation time, τ_r_) to be obtained—see [13,14] for example.

### 1.3. Scope of This Work

In this work, we plan to give illustrative examples of the use of time-resolved fluorescence, rather than an exhaustive review, based on our work to assess bioactives of plant origin in foodstuff. This will start with a “field” application through to bioactive extraction, storage, a domestic process (cooking) and the interaction of a bioactive with serum albumin; an important blood transport protein.

**Scheme 1:**
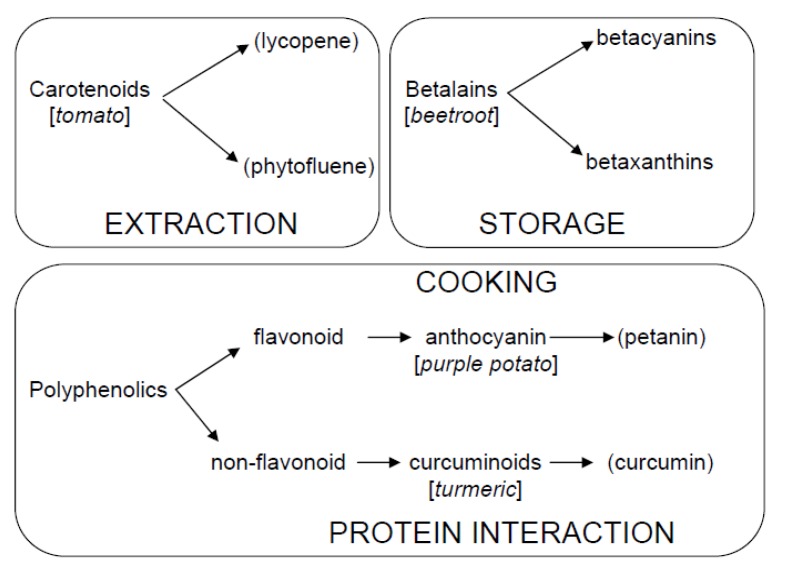
Principal compounds that will be addressed here, showing their [*origin*] and main (sub type) along with the APPLICATION area in this work.

Since polyphenolics are a major group, we will consider representative flavonoid and non-flavonoid phenolic compounds (anthocyanins [19] and curcuminoids [20,21]), see Scheme 1. Anthocyanins are most notably responsible for the red colouration in leaves and petals in trees and flowers. They are also encountered in fruit and vegetables and can be responsible for the bright colouration of these food products. Apart from their aesthetic, recent research has shown that anthocyanins (and other phenolics) have high free radical scavenging activity, anti-inflammatory and anti-microbial properties and it is suggested that their consumption can decrease the risk of chronic diseases, such as heart disease, type 2 diabetes and cancer [2,22,23]. Because of their potential antioxidant properties [23,24], anthocyanins have been promoted as dietary compounds (functional foods) and colouring in the food industry [25]. In this work, we make use of a purple pigmented potato (*Purple Majesty*—registered in the British Potato Variety Data Base as follows; (i) Parentage—All blue X ND2008-2; (ii) Breeder—San Luis Valley Research Center; (iii) Breeder’s agent– Albert Bartlett and Son Ltd., Airdrie, UK. This cultivar has been produced and marketed in the UK) as the source of anthocyanins, principally petanin [26,27], and assesses the effect of cooking on their form. In some plants, instead of anthocyanins, yellow to red colouration originates from indole derived betalains [28]. These pigments are found in *Caryophyllales* and yellow to orange colours are obtained from betaxanthins, with the red to violet colours stem from betacyanins. These compounds, extracted from beetroot (*Beta vulgaris*), have been studied using time-resolved fluorescence [29] and even suggested for use in dye-sensitised solar cells [30]. Here, we present a study on beetroot extract and assess the effect of storage condition using fluorescence.

As plants use photosynthesis to produce energy it is not surprising that chlorophyll fluorescence [31,32,33] may also need to be kept in mind when observing bioactives. Indeed, in food crops, the monitoring chlorophyll can give an indication of the plant health [34,35]. As well as chlorophyll, carotenoids are involved in the photosynthetic process and can channel energy (via absorption of light) into the chlorophyll [36,37] and have also been shown to act as in a photoprotection role [37]. The interest in the extraction and application of lycopene [38] in the food industry stems from its potential antioxidant behaviour to give health benefits [39,40,41], although its availability *in vivo* has been questioned and the health benefits obtained related to other metabolic products [42]. In this work, we consider the use of fluorescence to check if lycopene is affected when it is extracted with the aid of ultrasound. Lycopene is responsible for the red colouration in tomatoes. As tomatoes ripen the green, chlorophyll rich chloroplasts transform into carotenoid rich chromoplasts [43] giving rise to the characteristic red colouration. This would have an application when making use of the extracted lycopene as an additive for either enhancing or preserving foodstuffs.

Finally, we use fluorescence to study the interaction of non-flavonoid polyphenolics; curcuminoids obtained from turmeric, with the major blood transport protein serum albumin. Turmeric (*Curcuma Longa* L) is obtained from the rhizome of the *Zingberaceae* family and has a long history as an ingredient in cooking. The main extraction constituent, curcumin exhibits fluorescence and its photophysics are markedly affected by the polarity, hydrogen bonding and pH. This provides a means to monitor its interaction with proteins, which is important if its potential health role is to be fully investigated. Serum albumin, because of its transport role in blood, has been employed as a model to investigate the protein–curcumin interaction [44].

From these illustrative examples it is hoped to give an indication of the use of time-resolved fluorescence as a promising method by which to investigate food related bioactive compounds, based on a mini review of our work in this area.

## 2. Experimental Section

Since both the sample preparation and experimental details depend on the bioactive to be studied, the reader is pointed towards the original work [44,45,46,47,48] for a detailed description. Here, just the general common outlines will be given and since our emphasis is on the use of fluorescence, we will very briefly discuss some aspects. It should be noted that there is no real difference in sample preparation between the use of steady state or time-resolved methods. If performing a cuvette based measurement, with excitation source and detector at a right angle to each other, the sample solution should be optically diluted (absorbance ~0.1) to avoid absorption effects (of excitation/emitted light). If more concentrated solutions or solids require measurement then a “front face” measurement configuration is used. Details can be found in general fluorescence texts [13,14,15]. When measuring steady state fluorescence spectra, a correction for the wavelength response of detector/monochromator employed may be needed. In the case of time-resolved fluorescence measured using time-correlated single-photon counting, the major correction is that for the temporal response of the instrument. Ideally, the instrumental response (also known as the IRF or prompt) should be a delta function. However, in reality all instruments have a temporal response that can distort the measured fluorescence decay. The width of the instrumental response will depend on the equipment used, principally relating to the light source, detector and timing electronics. To account for this in the data analysis, the measurement of the instrumental response (usually with a scatter, e.g., for solution this can be colloidal silica) at the excitation wavelength, as well as the fluorescence decay, is required. The effect of the instrumental response can be addressed using numerical convolution. Applying reconvolution, where the convolution is applied to the kinetic model, before comparing to the measured data is advantageous over the more direct deconvolution method as this is more stable (without the loss of information associated if deconvolution was attempted) [16]. Using reconvolution analysis, it is generally accepted that fluorescence lifetimes ~10% of the instrumental response (at full width half maximum) can be resolved, with goodness of fit judged in terms of weighted residuals and reduced chi-squared value.

### 2.1. Steady State Measurements

Excitation-emission matrices and steady state spectra were obtained using a HORIBA Scientific FluoroLog 3, with the data treated using Fluoressence software. UV-VIS absorption measurements were performed on both Shimadzu UV-1800 and UV-1650PC spectrophotometers.

### 2.2. Time-Resolved Measurements

The measurements involving the acquisition of both lifetime and TRES (time-resolved emission spectrum) data were performed using a HORIBA Scientific DeltaFlex fluorescence lifetime system equipped with a PPD detector and DeltaDiode excitation sources. The TRES measurements, involved measuring the fluorescence decay for a set data collection time at a particular wavelength then incrementing the wavelength. Every fluorescence decay was recorded with the same collection period (over a specified wavelength range). This results in “3D” dataset of wavelength-intensity-time, which can then be globally analysed using the DataStation software as a sum of exponential components of the form;

(1) I(t)=∑i=1nαiexp(−tτi)

where τ is the fluorescence lifetime and α, the pre-exponential factor, which reflects the relative concentration present of that species. The fractional (*f*) or relative amplitude (expressed as %) of each fluorescing component given by

(2) $$f=\frac{\alpha_{i}\tau_{i}}{\sum \alpha_{i}\tau_{i}}$$

This relates to the contribution to the overall (steady state) fluorescence and is basically the pre-exponent weighted by the appropriate lifetime. An instrumental response function (IRF) or prompt was also recorded and from reconvolution analysis, decay associated spectra were also obtained (pre-exponential weighted by lifetime plotted against wavelength). The average lifetime was calculated as follows

(3) τ_ave_ = Σα_i_τ_i_

Kinetic TCSPC measurements were made using the DeltaFlex with the DeltaDiode excitation source operated at 100 MHz. In this mode, the minimum collection period can be as low as 1 ms and up to 10,000 sequential decays can be acquired, along with the instrumental response in order to perform reconvolution analysis. The datasets obtained were analysed in a batch mode using DAS6 software employing a multi-exponential model. From analysis of these data, the intensity (total counts in decay) and the average lifetime were returned. Graphing and analysis was performed using Origin 8 software.

High time-resolution measurements were performed on a HORIBA Scientific Ultima UltraFast system equipped with a microchannel plate detector close coupled to a CFD-2G amplifier-discriminator feeding into FluoroHub A+ timing electronics. The time resolution per histogram point was 307 fs, with 16 k histogram points recorded in each dataset. Excitation was made using DeltaDiode lasers operating at 20 MHz and giving an IRF (or prompt) with a full width at half maximum (FWHM) of 36 ps at 409 nm (DD-405L), 52 ps at 531 nm (DD-532LN) and 77 ps at 478 nm (DD-485L). The emission was monitored via a polariser at the magic angle and data were analysed by reconvolution fitting to the sum of exponentials, see Equation (1). As a “rule of thumb” it is often thought that during a lifetime, 10% of the IRF FWHM can be resolved, although it should be pointed out that when measuring very short-lived fluorescence the measurements are not trivial; both optical considerations and the stability of the equipment’s electronics are crucial.

### 2.3. Time-Resolved Fluorescence Microscopy

Optical examination of the samples was performed using a HORIBA Scientific DynaMyc equipped with a Lumenera Infinity 3-1C CCD camera to collect images using white light excitation (Lumin Dynamics X-Cite 120PC-Q), with emission selected via a beam splitter or filter cube. Intensity images in the blue, green and red spectral regions used the following filter sets (excitation/dichroic/emission); (370 nm/390 nm/460 nm), (500 nm/525 nm/545 nm), and (575 nm/600 nm/630 nm), respectively. The objective magnification was ×10.

### 2.4. Determination of Bioactive Components and Antioxidant Activity

The pH shift method adapted from Ribereau-Gayon and Stone Street (1965) was used to estimate the anthocyanin content in the samples of purple potato and has been previously employed in other works [49]. The absorbance of each sample at both pH <1.0 and pH 3.5 was measured in a spectrophotometer at 700 nm (which allows background correction) and 520 nm (to determine the anthocyanin content) against a blank. In order to obtain the absorbance (A) related to the total anthocyanins, the following equation was used

(4) A = (A_520_ − A_700_)pH_0.6_ − (A_520_ − A_700_)pH_3.5_

Considering the Beer-Lambert law, the concentration of total anthocyanins (g/L) can be calculated according to Equation (5).

(5) Anthocyanin concentration (g/L) = (A*MW)* ε^−1^*l^−1^

where, A is the absorbance (calculated from Equation (4)), MW is the molecular weight of a reference pigment (Cyanindin-3-glucoside)—449.2 g/mol, ε is the molar absorptivity (extinction factor 26,900 L·cm^−1^·mol^−1^), l is the optical path length in centimetres (1 cm). Then, and considering the dry and fresh weight (FW) of the different samples, the total anthocyanin content was calculated and expressed as mg Cyanindin-3-glucoside/kg FW.

The total quantity of phenolics was measured by the Folin-Ciocalteau method. Briefly, freeze dried potato was re-suspended in methanol:water (50:50, v/v). This solution was filtered and a sample added to a dilution of Folin-Ciocalteau reagent and distilled water. After 5 minutes Na_2_CO_3_ was added and the mixture was left in the dark, at room temperature, for 2 h. The absorbance of the solution was measured at a wavelength of 765 nm against a blank. The optical density (O.D) was compared to a standard curve (y = 0.001x; *R*^2^ = 0.998) prepared with 50 to 500 mg·L^−1^ of gallic acid and the result expressed as mg·L^−1^ gallic acid equivalents (GAE). Results are expressed as mg GAE = /100 g FW.

Antioxidant activity was determined using ferric reducing antioxidant potential (FRAP). In summary, dried purple potato was re-suspended in 80:20 (v/v) acetone/water. A sample of this was added to FRAP working solution and incubated in a water bath at 37 °C for 4 min followed by measurement of absorbance at 593 nm against a blank. The Optical density was compared to the standard curve (y = 0.4895x; *R*^2^ = 0.9931) for ferrous sulphate (FeSO_4_) solutions, with concentrations between 0 and 10.0 mM. Results are presented as Fe II (mM) produced/100 g FW.

## 3. Results and Discussion

### 3.1. In the “Leaf” (Kinetic TCSPC and TRES to Monitor Chlorophyll)

Although we do not consider it a bioactive, the fact that chlorophyll is present needs to be kept in mind and knowledge about its fluorescence behaviour understood. In relation to the food industry, the ability of producers to ascertain the health of plants in the “field” is becoming an economic necessity and remote sensing [50,51] has been employed, with an emphasis on monitoring chlorophyll fluorescence to monitor plant health [52,53,54]. Monitoring chlorophyll fluorescence has also been used to assess the influence of herbicide [55] and to this effect time-resolved fluorescence also employed [56]. Fruit ripening is also an area where monitoring the change in chlorophyll emission is useful and this has found application with tomatoes [57] and even post-harvest with olives [58,59]. Use of the chlorophyll absorption spectrum has also been proposed to monitor fruit ripening [54]. In fact, the whole subject field of photosynthesis and chlorophyll research is an exceedingly large area of investigation spanning from fundamental biology into materials and green energy applications. For the purpose of this article, we shall give two types of illustrative time-resolved fluorescence measurements applied to observing leaf fluorescence in general, rather than to any specific food crop.

**Figure 2:**
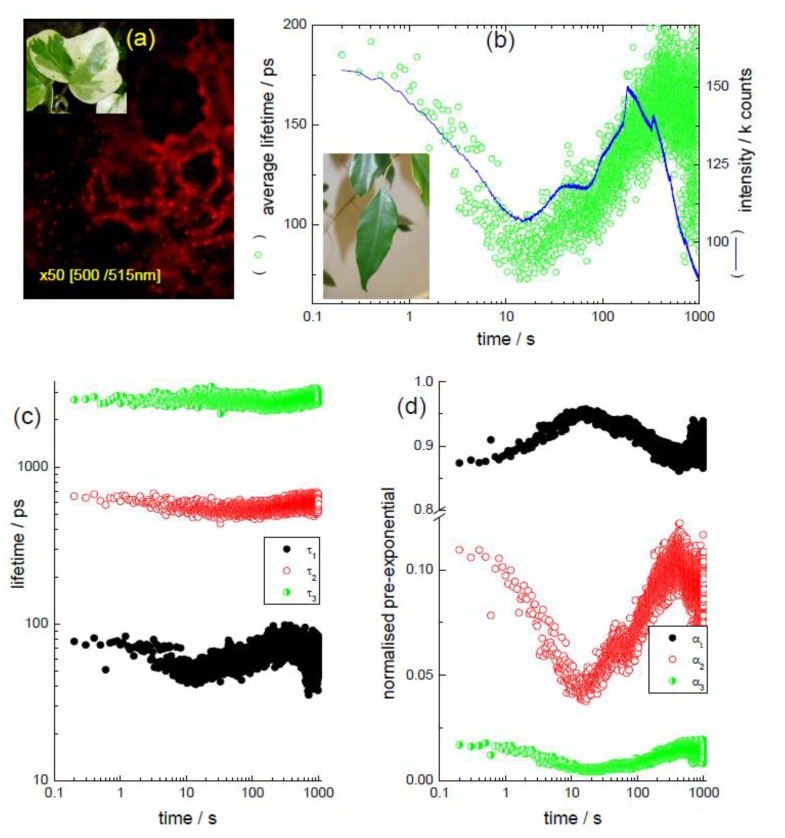
(**a**) Microscopy image, via filters of chloroplast emission from a leaf (*Hedera—*inset); (**b**) outcome of a kinetic TCSPC measurement showing both the variation of fluorescence intensity and average lifetime for a whole leaf (*Ficus*—inset). The excitation was at 478 nm and the emission was selected using a filter for large spectral coverage. Three exponential analysis of the kinetic TCSPC measurement, showing (**c**) the lifetime values and (**d**) their normalised pre-exponential contribution. Adapted from [45].

The photosynthetic chloroplasts can be easily observed using fluorescence microscopy and Figure 2 gives examples using two different plants. Figure 2a shows the red (steady state) emission of chloroplasts in a variegated leaf (*Hedera*). This image, although providing positional information (the chloroplasts can be seen boarding stomata), does not provide detail relating to any dynamic process that can be occurring, with the exception of perhaps repeating the measurement some time later and attempting to compare intensities. This may appear a simple measurement, but is in fact reasonably complicated as any change in the intensity of the excitation light would need to be accounted for, as would changes in the leaf position and photobleaching of the chloroplasts themselves. The repeat comparative measurements have suddenly become non trivial. A time-resolved fluorescence study on the other hand, in this case exciting at 478 nm (DeltaDiode-485L at 50 MHz) and monitoring one spot on a freshly picked leaf (*Ficus*) using a kinetic TCSPC measurement, can be employed. This form of measurement is not influenced by changes in the excitation intensity or photobleaching. Here, 10,000 fluorescence decays were collected sequentially with a data collection time per decay of 100 ms. The dataset was analysed as the sum of three exponential components and the average lifetime obtained. This is shown in Figure 2b along with the intensity obtained from the number of counts in the decay curves. It is obvious that the fluorescence signal from the chlorophyll is not constant with time. After the onset of the measurement (and the laser irradiation) over the first 10 s, there is a decrease in intensity, followed by a longer recovery to the initial values at ~10 min.

Changes in chlorophyll intensity have been ascribed to the Kautsky effect [35,60] and relates to the reduction of the number of electron acceptors in the photosynthetic pathway [31]. In a simple model, light absorbed by photosystem II (PSII) can go to drive the photochemistry of photosynthesis or be lost; either as fluorescence or heat [33]. The rates of these processes are in competition, with an increase in one leading to decreases in the other two. Although this measurement can be possible using the steady state intensity, as can be judged by the intensity given in Figure 2b, at longer times there is a difference between the trend in the intensity and the average lifetime, which can relate to photobleaching of the chloroplasts—an effect not seen in time-resolved data as they are independent of fluorophore concentration.

Furthermore, although Figure 2b displays the average lifetime, in order to obtain this, a fitting of the time-resolved data are required and for this dataset a model involving the sum of three exponential decays was employed, with data analysed in a “batch mode”; fitting each of the decays to this model (using reconvolution). The lifetimes obtained are given in Figure 2c, along with the related normalised pre-exponential factors (α_i_), indicating their relative concentrations in Figure 2d. Figure 2c shows that throughout the time course of the experiment the three lifetimes are more or less constant. The change in the average lifetime (see Equation (3)) is caused by differences in their relative contributions to the fluorescence. Both the pre-exponentials for τ_2_ (~540 ps) and the shorter-lived τ_1_ (~55 ps) appear to change the most; the longer-lived decay (~2.6 ns) is less affected. The presence of three lifetimes is not unsurprising, although there has been some discussion concerning their origin. For example, in a report using spinach [61] a “fast” decay time has been attributed to open PSII centres, a middle lifetime to Q_B_-non reducing centres (Q_B_ relates to the secondary quinine electron acceptor) and the longer (ns) decay to closed PSII centres, although determining the exact origins has led to debate [32]. The knowledge of lifetime values for different processes can also enable rate constants (k) to be determined (as τ = 1/k).

**Figure 3:**
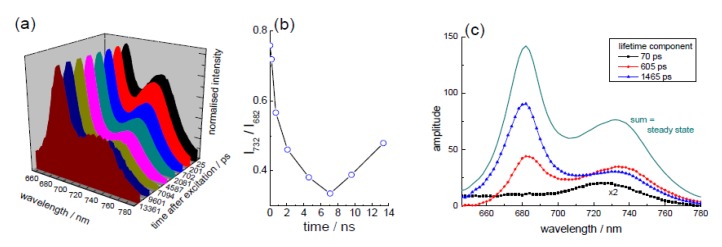
TRES measurement performed on a whole leaf (*Ficus*), showing (**a**) time-resolved emission spectra; (**b**) change in peak ratio of the principal emission bands with time after excitation and (**c**) decay associated spectra obtained via a global analysis of the TRES dataset. Adapted from [45].

To further explore these lifetime components a TRES measurement can be performed, taking time-resolved fluorescence lifetime measurements at wavelength increments for fixed data collection periods. This creates a dataset composed of time-wavelength and intensity (as shown schematically in Figure 1c). Taking “time slices” at different times relative to the excitation pulse allows the spectral shape at these times to be ascertained. For the *Ficus* leaf used here, representative “time slices” are shown in Figure 3a. The spectrum shape has peaks ~650 nm and ~730 nm, characteristic of chlorophyll emission from an intact leaf [60]. Although the relative intensity of these bands can depend on excitation wavelength [62], interestingly their ratio appears to display a time dependency, as can be seen in Figure 3b. A further way to analyse this dataset is to perform global analysis, determining common lifetimes and obtaining their contributions at each wavelength. From this analysis, it is possible to obtain decay associated spectra (basically the pre-exponential factor weighted by its lifetime, plotted against wavelength). These are shown in Figure 3c and overall the data are in keeping with the longer wavelength band, which exhibits the shortest lifetime, relating to photosystem I (PSI) emission [60,63,64]. The shorter wavelength emission is associated with PSII [63], although a longer wavelength band (slightly longer than that seen for the 70 ps spectrum in Figure 3c) is also apparent, which has also been ascribed to PSII [60]. It is not the purpose of this article to go into great detail of the mechanisms involved, but to demonstrate the ability of time-resolved fluorescence to be used as a tool to help uncover them, which in relation to chlorophyll fluorescence, is it very adept. It is clear that it is possible to enable the time dependent contribution from the different photosystems and overall kinetic information to be obtained, which would not be as simple if employing steady state fluorescence.

### 3.2. Extraction of Lycopene from Tomato Pulp Using Ultrasound (Decay Associated Spectra and Lifetime Determination)

Largely because of its antioxidant potential, there is an interest in using lycopene and its extraction from tomatoes as an area of investigation. “Green” extraction techniques for carotenoids, such as supercritical extraction [65] and the use of ultrasound treatment [66,67], have attracted attention as means by which to obtain carotenoids. Relatively low ultrasound frequencies (24 kHz) appear benign to carotenoids [68], including lycopene [69]. Here, we will make use of a higher frequency of 583 kHz [48]. The biosynthetic pathway involved in producing lycopene contains many intermediates, generally considered starting with geranylgeranyl pyrophosphate and proceeding to lycopene, which can be further involved in the formation of cyclic carotenoids [5,70,71,72,73]. Carotenoids are widespread pigments found in nature and in plants and can be involved in the photosynthetic process [37,74,75]. As tomatoes ripen, they go from green (chlorophyll rich) to red (lycopene rich) colour, which in physiological terms relates to chlorophyll containing chloroplasts transforming into chromoplasts [76] containing carotenoids. This means that there is the possibility that any extract from tomato would contain precursors to lycopene as well as chlorophyll, which would also be present if the tomato was damaged [57]. Here, we employ the measurement of time-resolved emission spectra to obtain the fluorescence decay parameters from the tomato extract in hexane. These data are compared to that of pure lycopene in hexane, which entails the use of high time-resolution TCSPC for the acquisition of very short-lived decays. This type of measurement is not trivial and requires good timing stability and care with the optical set up.

**Figure 4:**
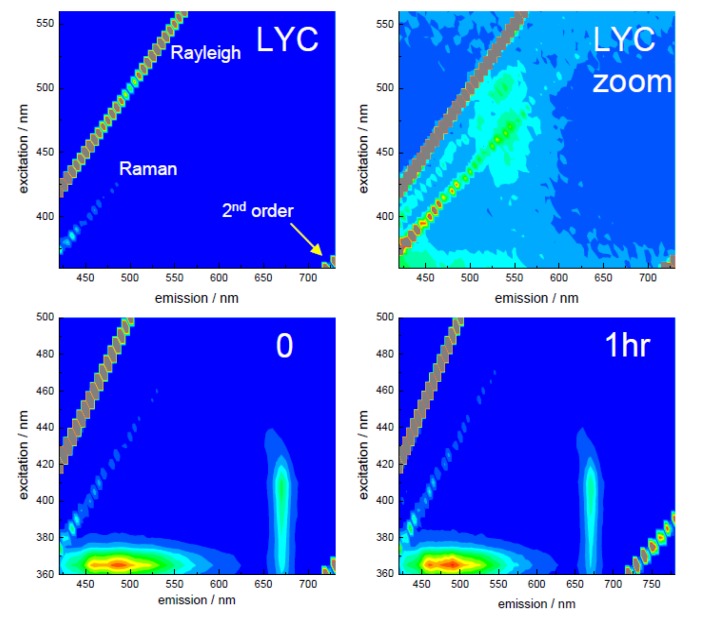
Fluorescence excitation-emission spectra (EEM’s) for the samples, showing excitation wavelength (*y*-axis) emission wavelength (*x*-axis) and the intensity as rainbow scale (red—high, blue—low). The lycopene EEM (LYC) has also been scaled (LYC zoom) to show the lower intensity emission at ~550 nm. The scattering positions relating to Rayleigh, Raman and second order are also indicated. Adapted from [48].

Prior to the time-resolved measurements, excitation-emission matrices (EEM) were recorded to ascertain suitable excitation and emission wavelengths, as well as any influence of scattering (Rayleigh and Raman) with these low quantum yield samples. These are shown in Figure 4 for lycopene (LYC), untreated (0) tomato extract and extract ultrasound treated for 1 h (1 h). Since no masking was applied, diagonal lines are seen relating to the presence of both Rayleigh and Raman scattering. Scaling the LYC EEM enables the relatively low level of fluorescence emission around 550 nm, expected from lycopene [77], to be seen. There have been reports of emission bands at 510 nm, 543 nm and 581 nm [78] in the spectral region used in this study. Considering the EEM’s from the tomato extract (0 h, 1 h), again both Rayleigh and Raman scattering can be discerned. However, it is difficult to observe any emission related to lycopene. Instead, there are emissions close to 500 nm (with shorter wavelength excitation) and 670 nm, The shorter wavelength emission is consistent with the presence of phytofluene, with the excitation coinciding with the absorption peaks [48] and there are reports of its emission at this wavelength, although this can be affected by both solvent and temperature [74,79]. The longer wavelength emission can be assigned to the presence of cholorphyll [43,57,60]. The presence of these components is hardly unsurprising; phytofluene is produced in a step of the biosynthesis of lycopene, occurring after the production of phytoene from geranylgeranyl pyrophosphate [70,72], while chlorophyll is found in immature or damaged tomatoes [43,57].

**Figure 5:**
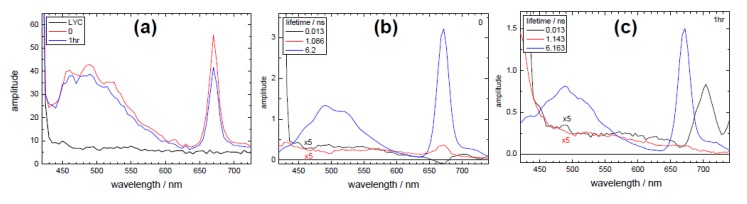
Results of TRES measurements, showing (**a**) the equivalent steady state spectra and the decay associated spectra for (**b**) the untreated extract and (**c**) extract treated for 1 h. Excitation was at 378 nm. Adapted from [48].

TRES measurements were performed on these samples, with an excitation wavelength of 378 nm as this could excite several of the principal constituents. Fluorescence decay measurements were taken at 4 nm increments and the resulting datasets analysed globally, with decay associated spectra obtained from this analysis shown in Figure 5. In all cases, the sum of three exponential decay components were required to fit the data, with one lifetime fixed at ~13 ps to account for any fast (e.g., scattering) process. The equivalent steady state spectrum, Figure 5a, shows an emission round 500 nm for both extracts (treated and untreated), which is absent in the lycopene sample, as is an emission peak at 670 nm. Considering the decay associated spectra for the extracts (Figure 5b,c), the two shorter-lived components contribute a small amount to a broad fluorescence emission, although the shortest-lived also shows a well-defined spectral peak at ~704 nm. The major emission relates to a lifetime of ~6 ns and shows features where both phytofluene (~500 nm) and chlorophyll (670 nm) are emitted. This lifetime is similar to that found for chlorophyll in solution [80], which it should be noted is longer than that observed in whole leaves [63]. It can be seen, considering the longer-lived decay associated spectra, that the ratio of the phytofulene and chlorophyll peaks is different for the untreated (0 h) and treated (1 h) samples. A possible explanation is that there has been a change in the relative contributions of photosystem II (PSII, emitting toward 670 nm) and photosystem I (PSI) chlorophyll that emits at longer wavelengths [60,63]. This is also evident when looking at the relative contributions of the ~1 ns decay associated spectrum. These results may be interpreted that upon ultrasound treatment there is an increase in the relative emission relating to PSI or conversely, and most likely, emission from PSII has reduced.

**Figure 6:**
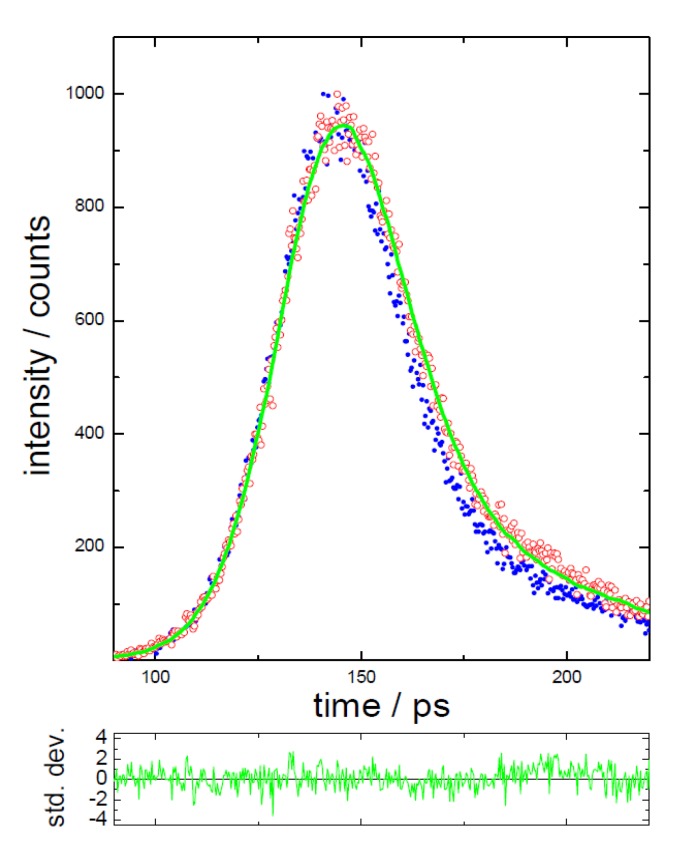
Representation of the decay of lycopene in hexane (red), also showing the IRF (of 36 ps—blue), fitted function (2 exponential green) and weighted residuals (green), close to the peak of the decay. The excitation wavelength was 409 nm (DD-405L) and the emission at 550 nm. Adapted from [48].

If we turn to observing the, admittedly, low level of fluorescence coming from the lycopene itself, the use of high time-resolution TCSPC equipment is required. An excitation wavelength of 409 nm was selected, although the absorption of lycopene is more intense towards longer wavelengths. Principally, because of the low quantum yield exhibited by this sample, excitation at a longer wavelength (more into the main absorption band), with the emission monitored at 550 nm would lead to the possibility of detecting Raman scattering (both from dissolved compounds and solvent), as can be seen in the EEMs in Figure 5. 550 nm is an emission wavelength that others [78] have observed fluorescence, although in our extract we may expect emission from other species at this wavelength as well. Previous studies on lycopene have reported short-lived emission [75,78] and a value of 4.7 ps quoted when using hexane as a solvent [81]. This value is at the limit of that achievable with the time-correlated single-photon counting technique employed here and the use of the laser wavelength selected also processes a narrow optical pulse (IRF of 36 ps). This is important in order to attempt this type of measurement. An example of a fit (to 2 exponentials) to the decay of lycopene in hexane (LYC) is shown in Figure 6, focussing on the peak region, with the decay parameters for all samples given in Table 1.

**Table 1:** Time-resolved decay parameters obtained using reconvolution analysis, with excitation at 409 nm and emission at 550 nm. Note, if comparing the normalised pre-exponentials (α_i_) then τ_1_ accounts for practically all the emission.

Sample	Lifetime/ps			Fractional/%		
	τ_1_	τ_2_	τ_3_	*f*_1_	*f*_2_	*f*_3_
LYC	5.1 ± 0.8	807 ± 20		84	16	
0	4.4 ± 0.4	313 ± 69	2014 ± 183	62	8	30
1hr	5.1 ± 1.1	277 ± 57	1758 ± 123	62	7	31

The Table 1 data show that in all cases the decay was dominated by a very short-lived decay (α~1) of the order of 5 ps. This compares favourably to the 4.7 ps previously observed [81] and calculated [78] for lycopene. However, it should again be stressed that these measurements are far from trivial and because of control of measurement conditions (optical/run times/collection rates) this dataset should at least show relative differences. The lycopene control (LYC) requires a small amount of an additional component (807 ps) to give an adequate fit; note that this was commercially obtained with a stated purity of ~90%. For the extracts, the sum of three exponentials were required and it should be kept in mind that the values obtained are consistent with those from the TRES taken on a lower time-resolution equipment (Figure 5); with a better resolution of the shorter-lived component at the expense of the longer-lived ones (only present in minute amounts in this analysis). There is the possibility that the other decay times may relate to impurities or the presence of other compounds in the samples. Although, in light of the previous data, it appears that on this time-scale it is not possible to distinguish any significant difference between the ultrasound treated and untreated samples. Thus, it would be tempting to suggest that the ultrasound extraction treatment does not have any significant effect on the lycopene itself, but has more of an influence on chlorophyll present in the tomato sample.

### 3.3. Effect of Storage on Betalains in Raw Vacuum Beetroot (Lifetime Determination of Betalains)

Betalains are nitrogen containing pigments that replace anthocyanins in plants and fruits, mainly in the families of the *Caryophyllates* [6]. Betalains have application as food colourings and recent interest has focused on their antioxidant potential [28]. The storage of food products is clearly an important area as storage length can affect the quality and availability of foodstuffs, while conditions and packing can interfere with the product’s shelf life and composition [82,83,84]. The type of packing involved is dependent of the product to be stored, but a major application is the removal of oxygen to prolong storage and to reduce spoilage of food. Time-resolved fluorescence has been applied to study the presence of oxygen in food packaging, although it can be considered an expensive approach [85].

For the context of this work, we will illustrate how time-resolved measurements can be directly applied to the study of raw beetroot that has been stored under vacuum (at refrigeration temperature) for up to 41 days. Raw beetroot was placed into bags and oxygen removed by vacuum, with the samples stored at refrigeration temperatures. Samples were removed at pre-determined time intervals, from the packaging and then freeze dried. Prior to measurement, these samples were resuspended in a (50:50, v:v) methanol: water mixture and comparison made to the original (non vacuum freeze dried—control) sample using high time-resolution fluorescence spectroscopy.

**Figure 7:**
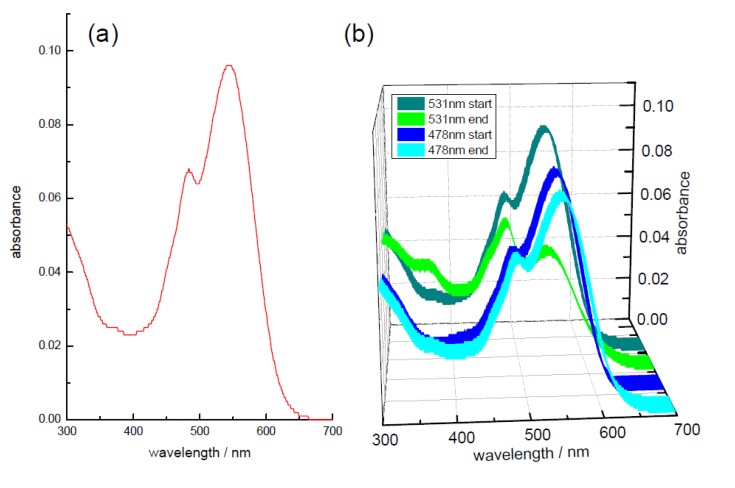
(**a**) absorption spectrum for a freshly made solution of the sample stored for 41 days in (50:50, v:v) methanol: water; (**b**) effect of laser irradiation during the time-resolved measurements, either at 531 nm or 478 nm on the same sample.

**Figure 8:**
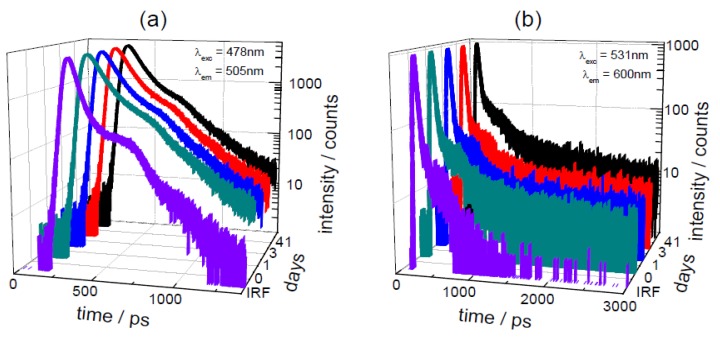
Time-resolved decays for the beetroot samples stored for different times; (**a**) using 478 nm excitation; (**b**) with 531 nm excitation. The IRF in both cases is also shown (FWHM of 77 ps at 478 nm and 52 ps at 531 nm).

An illustrative absorption spectrum from a sample stored for 41 days is given in Figure 7a. This shows the presence of a main peak at 544 nm with another band situated at 483 nm. These are similar to a previous report [30], with a combination of betaxanthin (shorter wavelength) and betacyanin (longer wavelength) in the extract. Although beetroot is a main source of the betacyanin, betanin [28], the betaxanthin, indicaxantin, is also present [28]. This has been shown to have viscosity dependent photophysics [86], with an emission around 505 nm to 520 nm in solution [86,87]. This appears to be the major emission observed from beetroot extract [29], although after separating indicaxantin and betanin from beetroot extract, a longer emission at ~608 nm from the betanin (when excited at 535 nm) can also be observed [30]. For the time-resolved study, we selected beetroot samples which had been resuspended in the (50:50, v:v) methanol: water mixture after freeze drying. Prior to freeze drying the samples had been stored in vacuum packaging for different times. The original “parent” sample, which was not subjected to vacuum packaging and freeze dried at the same time as the other samples were packaged, was measured for comparison. As the extract contains a combination of betaxanthins (shorter wavelength) and betacyanins (longer wavelength) two excitation and emission wavelengths were used (478 nm and 505 nm; 531 nm and 600 nm) to probe both of the main absorption bands and the decays (along with IRF) are displayed in Figure 8. The time-resolved parameters obtained from the analysis of these measurements are given in Table 2.

Before concentrating on the time-resolved data, it should be pointed out that during the experiment using the 41 day sample with 531 nm excitation, it was noted that although similar concentration solutions were used the length of time to acquire the data was considerably longer. Comparison of the initial absorption spectrum before the measurement, to that taken at the end of the experiment (see Figure 7b) showed a marked (~50%) decrease in the longer wavelength band intensity. This type of behaviour was not seen when exciting at 478 nm with this sample and no such dramatic decrease was noted in the other samples when exciting at 531 nm. Thus, it appears that the storage condition may have affected the photostability of the betanin. As the lifetime measurement is independent of concentration, this decrease in the amount of the betanin should not affect the values obtained. The results for the excitation wavelength of 478 nm appear consistent for all of the samples. The dominant decay contribution to the overall (steady state) emission (*i.e.*, fractional, f_2_, contribution) is from a lifetime of ~70 ps, although in actual concentration terms (as signified by the α_i_) there is more of the shortest-lived (~15 ps) lifetime present. In these samples, the main emission would be expected to come from indicaxananthin, which when purified has been reported to have a biexponential decay with a short-lived component between 6.5 ps and 14.1 ps and another (major contribution in terms of fractionals) ranging from 27 ps to 161 ps, depending on solvent [86]. Although we have had to employ an extra exponential, the values that we obtain are of a similar magnitude to those reported [86] and it should be remembered that we are using an unpurified beetroot extract in a solvent mixture. Also, for the purpose of this work we are looking for relative differences between the samples, which when considering the 478 nm excitation wavelength no significant difference can be determined.

**Table 2:** Time-resolved decay parameters for stored raw vacuum beetroot samples obtained from fitting to Equation (1) using reconvolution analysis.

days	λ_exc_	Lifetime/ps				Pre-Exponential α_i_ [f _i_/%]		
stored	/nm	τ_1_	τ_2_	τ_3_	<τ>	α_1_ [f_1_]	α_2_ [f_2_]	α_3_ [f_3_]
0	478	15 ± 2	72 ± 2	264 ± 8	34	0.70 [31]	0.28 [59]	0.02 [10]
1	478	16 ± 2	69 ± 2	265 ± 8	34	0.70 [31]	0.29 [59]	0.01 [10]
3	478	14 ± 2	70 ± 2	259 ± 7	33	0.71 [31]	0.28 [59]	0.01 [10]
41	478	14 ± 2	73 ± 2	300 ± 7	33	0.72 [31]	0.26 [57]	0.02 [12]
0	531	8.6 ± 0.6	549 ± 37		10.0	1.00 [85]	0.00 [15]	
1	531	8.4 ± 0.6	468 ± 39		9.6	1.00 [87]	0.00 [13]	
3	531	8.0 ± 0.7	501 ± 45		9.2	1.00 [86]	0.00 [14]	
41	531	7.7 ± 0.9	195 ± 15	1141 ± 67	14.1	0.98 [54]	0.01 [19]	0.01 [27]

Exciting at 531 nm and monitoring at 600 nm should favour the detection of betanin and in our data the dominant (both in terms of contribution to the steady state emission and concentration of that species) emission comes from a short-lived decay of ~8 ps. It is only for the 41 day sample that any difference is seen in the recovered parameters, with the need for an additional longer-lived component to model the decay. This coupled with the previous observation of potential photobleaching, as evident by a reduction in the betanin absorption band, is indicative of changes occurring within the longest stored sample that affect the photophysics of the bioactive constituents. It is clear that fluorescence techniques are applicable for this study and that with further structural information it may be possible to further elucidate and characterise the processes occurring. However, for the purpose of this work we will concentrate on illustrating the use and application of the fluorescence measurements.

### 3.4. Effect of Cooking on Anthocyanin and Antioxidant Activity (Decay Associated Spectra)

**Scheme 2:**
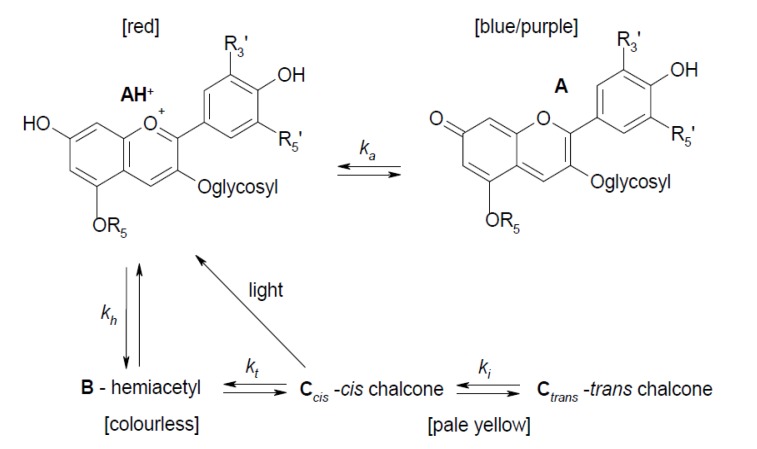
Relationship between the different anthocyanin (**AH^+^**, **A**, **B**, **C***cis*, **C***trans*) forms adapted from [100] and [colour] indications from [99].

One of the most common domestic processes that foodstuff containing bioactive materials can be subjected to is cooking. Here, we illustrate this using a purple pigmented potato (*Purple Majesty*), whose colouration is related to the presence of anthocyanins [88,89,90]. Several types of anthocyanin may be present and this is dependent on the variety of purple potato [26,91]. In the cultivar used here, the dominant anthocyanin is petanin [26,27], which although associated with tubers is also a purple flower and stem pigment [92]. Potatoes also contain non-anthocyanin polyphenols [24] that can complex with anthocyanins [93,94]. It is known that the cooking process can detrimentally interfere with the amount of anthocyanins in a product, as shown by recent studies using pigmented potatoes [95,96,97,98]. The stability of anthocyanins is dependent of their environment and they can exist in various forms, shown generally in Scheme 2, adapted from other work [99,100]. The characteristic highly coloured (**AH^+^**) form is usually only stable in highly acidic conditions and acidified solvents are commonly employed in the extraction of anthocyanins [19]. At lower pH values, the **AH^+^** form should be stabilised, while at the highest value it is expected that the anthocyanins are converted to via the **B** to **C** forms [101], with the potential that these forms can irreversibly degrade into an aldehyde and phenolic acid [19]. Although it should be noted that petanin has been shown to remain coloured in slightly alkaline solutions [102] and hence the use of water is valid for comparison as it both represents, in relation to food, the usual cooking medium and does not “force” via pH the formation of one dominant form. Here, we shall assess the effect of four types of cooking techniques (bake, boil, microwave, steam) in comparison to uncooked potato in terms of total phenolic and anthocyanin contents, as well as overall antioxidant activity, and see if this relates to the fluorescence behaviour of the sample.

**Figure 9:**
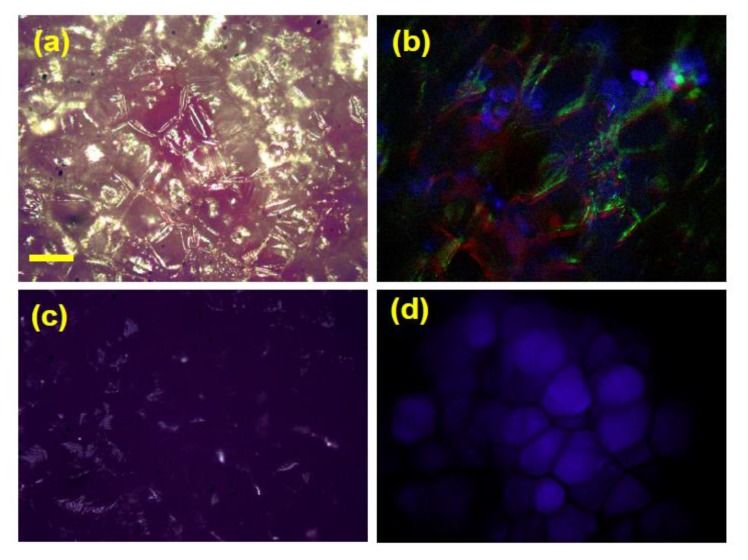
Fluorescence camera intensity images with white light illumination of a free hand slice of raw (**a**,**b**) and microwaved (**c**,**d**) *Purple Majesty*; (**a**,**c**) taken using a beam splitter, (**b**) R,B,G composite picture; (**d**) using blue filter cube. The bar (given in (a)) represents *ca*. 200 μm. Adapted from [47].

In *Purple Majesty* the anthocyanins are not found to be distributed in every cell, but restricted in location, as can be seen in Figure 9a, in which pigmented cells are visible along with the more common colouration for potato cells. Making use of colour filters and the natural fluorescence from the potato, it is also possible to obtain further details [47]. Figure 9b shows a composite image combining blue, green and red spectral regions and enables the differentiation of starch granules within the tuber along with the cell walls. The size of the granules is in keeping with other confocal microscope studies [103,104]. Upon microwave cooking, the coloured cells become present throughout the field of view (Figure 9c) as the cells are disrupted; spreading the anthocyanin. Also, it is interesting to note that the starch granules are no longer defined and appear to fill the cell structures (Figure 9d). This can be attributed to the heating produced by the microwave process as the starch gelatinised and swelled, as observed in fried potatoes [103].

**Figure 10:**
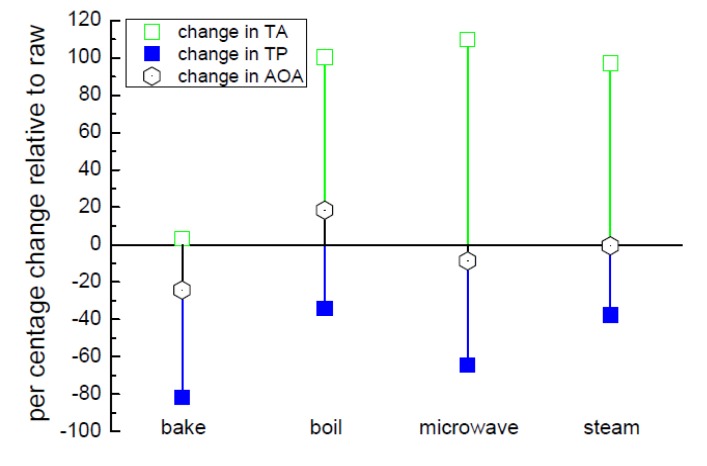
Change in bioactive content in purple potato, relative to uncooked, seen using different cooking methods. TP—total phenolic compounds, TA—total anthocyanins and AOA—antioxidant activity. Adapted from [46].

Analysis of the bioactives upon cooking (measurements on freeze dried, resuspended samples [46]), showing the relative (compared to raw potato) change in the totals of the phenolic and anthocyanin compounds along with the antioxidant activity in depicted in Figure 10. Overall, there is an increase in the amount of anthocyanins (expressed as Cyanindin-3-glucoside, C-3-g), when compared with the raw potatoes. It should be noted, however, that the level of anthocyanins in baked potatoes was not too different to that present in raw potatoes. Decreases are observed in both total phenolics and antioxidant activity (with the exception of boiling). These results are explained in detail in another work [46] and here we will now explore the use of time-resolved fluorescence to assess these samples. Anthocyanins can exist in different chemical forms depending on their environment, especially in terms of pH [94,102], which affects their stability. Although, complexation with other polyphenols can also produce a strong colouration [94]. Work using pure anthocyanins and models have shown that the “**A**” form emits at a longer wavelength than the “**AH^+^**” and is associated with a shorter decay time [105,106].

**Figure 11:**
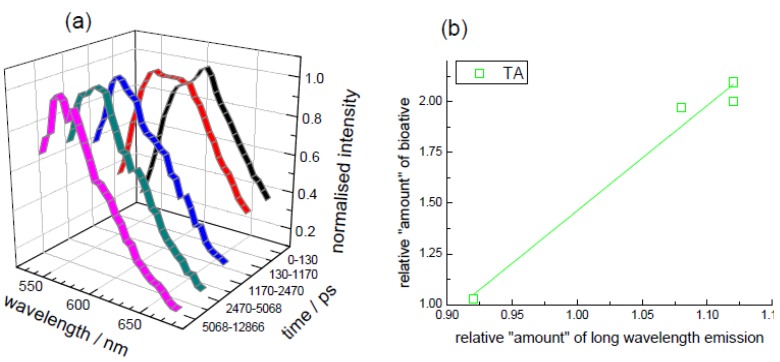
(**a**) TRES “time slices” measured from a sample of baked potato. The intensities are shown normalised. Excitation was at 510 nm (DD-510L); (**b**) Comparison of longer wavelength emission [46] with relative (to uncooked) quantity of TA. Adapted from [46].

Performing a TRES measurement (exciting at 510 nm) and taking “time slices” enables the spectral shape to be monitored with time. An example is given in Figure 11a for potato that has been baked. This shows evidence for (at least) two emissions; one at a longer wavelength prevalent at shorter times after excitation and another at shorter wavelengths, which dominates at longer times. It is also possible to perform spectral decomposition; allowing the longer and shorter wavelength emissions to be separated [46]. Plotting the relative contribution of the longer wavelength (shorter-lived) emission against the relative (normalised to that in the uncooked potato) amount of the total anthocyanin is shown in Figure 11b. This appears to indicate that the contribution of the shorter-lived, longer wavelength emission relates to the total anthocyanin content (determined chemically). A relation between fluorescence and the total antioxidant activity was not forthcoming. The use of TRES measurements (and decay associated spectra) can provide a wealth of information as both spectral and time data can be accessed. In this case, because the anthocyanin can exist in different forms [94,102,105,106] there is also the possibility to obtain information concerning their microenvironment from this type of measurement. This can either just be used to assess relative differences, or in the light of further information concerning the sample used to ascribe the spectra/decay times to different chemical forms. In this present work, we restrict ourselves in demonstrating the potential of these measurements and their application to monitor this kind of bioactive during an example of a domestic process.

### 3.5. Interaction of Curcuminoids with Serum Albumin (Kinetic TCSPC)

Turmeric extracts are principally composed of curcuminoids, which are non-flavonoid polyphenolic compounds. The dominant form in the extract is curcumin and its properties have been the focus of investigation because of its biological activity [20], which includes anticancer potential [107] and antioxidant behaviour [108]. Although it can appear a promising compound, with low toxicity, there is a limitation in relation to its bioavailability [109]. The fact that curcumin fluoresces has led to this phenomenon being used to investigate its behaviour in solution, with proteins and in cells [110]. Studies have concentrated on the role of hydrogen bond formation and making use of different solvents to study the curcumin photophysics [111,112,113,114,115], including ultrafast spectroscopy [116,117]. Serum albumin, because of its presence and transport role in blood, has been employed as a model to investigate the protein–curcumin interaction [118,119,120]. In this work, we employ the photophysical behaviour of curcuminoids to monitor their binding to serum albumin in solution. This is possible since the fluorescence in aqueous solution is highly quenched (also there is negligible solubility of curcumin in water) and upon binding becomes more fluorescent (an increase in both quantum yield and lifetime). A kinetic TCSPC measurement enables the use of the fluorescent lifetime as a means to monitor the binding process and to establish rate constants. This method is advantageous over steady state fluorescence, since these compounds can also photobleach (leading to decrease in emission intensity that can influence the kinetic signal) and has the sensitivity advantage over the use of UV-vis absorption.

**Figure 12:**
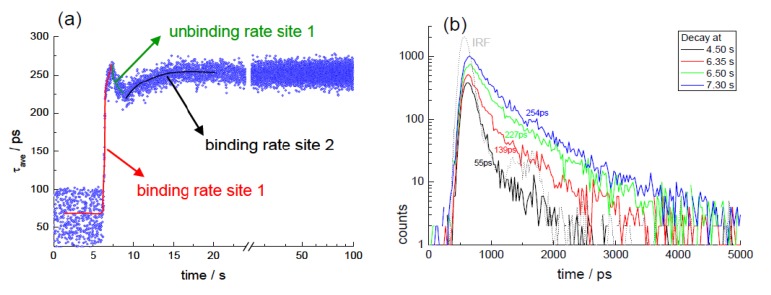
Binding of turmeric extract with serum albumin; (**a**) outcome of a two exponential analysis from a kinetic TSCPC dataset. 10,000 decay histograms were collected, each with an acquisition time of 10 ms; (**b**) representative decay histograms at different times at the beginning of the binding process. Adapted from [44].

To monitor the binding kinetics, a very high excitation rate (100 MHz) coupled with low dead time (<10 ns) timing electronics was used in order to efficiently acquire the fluorescence signal. The data collection time per decay was 10 ms and 10,000 decays were collected sequentially. Concentrated BSA in PBS (100 μL) was injected into a cuvette containing the extract in DMSO/PBS (2 mL) a few seconds after commencing the measurement. The mixture was continuously stirred and maintained at 20 °C. The outcome of the analysis from the measurement, exciting at 409 nm and monitoring the emission at 530 nm, is given in Figure 12a. This shows a sharp increase followed by a minor decrease, after which a slower increase and plateau occurs. This is consistent with a stopped flow study, which observed the presence of two kinetic steps giving rate constants in the order of 3–8 s^−1^ and 0.1–0.3 s^−1^, which were found to be concentration dependent and related to (at least) two binding sites [121]. From the analysis of our data, the fast process returns a rate constant of 5.11 ± 0.28 s^−1^, while the slower process gives a rate constant of 0.25 ± 0.02 s^−1^, consistent with the stopped flow experiment. In fact, the decrease that we observe may relate to an unbinding process as an equilibrium is established at the first site. The quality of the data obtained is given in Figure 12b and clearly illustrates the increase in the fluorescence lifetime upon addition of the protein. This is consistent with the fluorescence intensity, quenched in the aqueous environment increasing upon interaction with the BSA. The absorption spectrum was measured at the end of the experiment and the concentration of the turmeric extract (based on the extinction coefficient of curcumin in BSA [121]) estimated as ~ 8 × 10^−6^ M. Thus, the final molar ratio used was ~1:2 (BSA: turmeric extract), which should allow for an occupation of the binding sites with little unbound extract in solution.

It is also possible to again use the TRES/decay associated spectra approach to gain information concerning the binding environment, since the spectral position and lifetime of the curcuminoids are sensitive to changes in local microenvironment. This type of analysis has reinforced that the behaviour seen in the kinetic study is consistent with the idea of the presence of two binding sites, one surface site and an interior one, each with their own binding rate [44]. This demonstrates that additional information can be gained by making use of the sensitivity of the time-resolved fluorescence approach.

## 4. Summary

In this paper, we have attempted to illustrate how different time-resolved fluorescence techniques can be employed in the study of bioactive compounds related to the food industry. The results shown have in the main been based on results that we have published or are in the process of publishing. It is not intended to be an exhaustive article, going through all forms of fluorescence measurement (fluorescence anisotropy, for example, has not been utilised here) and all of the bioactives. Instead, we have intended to use illustrative measurements in order to show the potential of this technique, which has the advantage of giving an optical, non-destructive measurement, ideal for looking at molecular interactions on the nanoscale and providing an absolute measure, independent of concentration. The advantages of using fluorescence lifetimes were once thought of as the preserve of instrumentation centres, but are now becoming more accessible and easy to use. Thus, the measurement of time-resolved fluorescence is finding more use in a wide variety of research laboratories.
